# 'Does HPV affect my fertility?' Reproductive concerns of HPV-positive women: a qualitative study

**DOI:** 10.1186/s12978-021-01126-7

**Published:** 2021-04-01

**Authors:** Kowsar Qaderi, Seyedeh Tahereh Mirmolaei, Mehrnaz Geranmayeh, Farnaz Farnam, Shahrzad Sheikh Hasani

**Affiliations:** 1 Reproductive Health Department, School of Nursing and Midwifery, Tehran University of Medical Sciences, Eastern Nosrat St. Tohid Sq., 141973317 Tehran, Iran; 2 Gynecology Oncology Department, Imam Khomeini Hospital Complex (IKHC), Tehran University of Medical Sciences (TUMS), Tehran, Iran

**Keywords:** Reproductive health, Qualitative research, Fertility, Female, Warts, Human papillomavirus, Iran, Papillomavirus infection, Pregnancy outcome, Sexually transmitted diseases

## Abstract

**Background:**

Reproductive health changes can occur following infection with Human papillomavirus. HPV is the most prevalent sexually transmitted infection causing a variety of clinical manifestations ranging from warts to cancer. This study aimed to explore the reproductive concerns of women infected with HPV.

**Methods:**

In this qualitative study, we used the conventional content analysis approach, with the aid of MAXQDA.10 software, to analyze data extracted from the face-to-face semi-structured interviews with 20 Iranian HPV-positive women (sampled by maximum variation purposive sampling). The accuracy of this research was ensured according to the four criteria proposed by Guba and Lincoln.

**Results:**

Exploring participants' reproductive concerns, three main categories were identified from the interviews including concerns about fertility potential, pregnancy and non-pregnancy reproductive issues. HPV-positive women concerned about reduced female/ male fertility due to HPV, the impact of the HPV on the fetal health, adverse pregnancy outcomes such as miscarriage and preterm delivery, and mother-to-child transmission of HPV during breastfeeding. HPV-positive women with abnormal cytology results were anxious that becoming pregnant or taking hormonal contraception might worsen their abnormalities. Most married women were reluctant to use a condom. Participants requested further information about the potential reproductive risks of the HPV vaccine. They also wanted to know about the safety of HPV vaccine during pregnancy and breastfeeding.

**Conclusions:**

HPV-positive women had some reproductive concerns that should be considered in the designing of educational-consulting interventions. Women need to be better understood and informed about the impact of HPV on their reproductive health. Health care providers may lack knowledge about these specific areas, and they could benefit from additional up-to-date information to address women's reproductive concerns.

## Plain English summary

HPV is the most prevalent sexually transmitted infection among men and women of reproductive age worldwide. The role of HPV in cervical cancer is well known. HPV infections are correlated substantially with multiple reproductive system abnormalities. HPV can be a threat to the reproductive health of patients. Reproductive health refers to “a state of complete physical, mental, and social well-being and not merely the absence of disease or infirmity in all matters pertaining to the reproductive system and to its functions and processes, as defined by the World Health Organization (WHO) and the United Nations Fund for Population Activities (UNFPA).

In this qualitative study, we conducted face-to-face semi-structured interviews with 20 Iranian HPV-positive women to explore their reproductive concerns. The conventional content analysis approach, with the aid of MAXQDA.10 software, was used to analyze data extracted from the interviews.

HPV-positive women identified some reproductive concerns such as worrying about reduced male and female fertility potential, the impact of HPV on fetal health, negative pregnancy outcomes (miscarriage and preterm delivery), and the safety of breastfeeding. HPV-positive women who had abnormal cells in their cervical cytology results were anxious that becoming pregnant or taking hormonal contraception might worsen their health condition. Most participants were reluctant to use a condom in spite of being recommended to use it. Women also asked about the potential reproductive risks of the HPV vaccine. HPV-positive women need to be better understood and informed about the impact of HPV on human reproductive in educational-consulting interventions.

## Background

HPV is the most prevalent sexually transmitted infection among men and women of reproductive age worldwide [1], and its effect on cervical cancer is well known [2]. HPV testing reaches the maximum level of accuracy in cervical cancer screening [3].

HPV infections are significantly associated with reproductive function abnormalities [1]. In comparison to studies related to HPV oncogenic effects, there is a lack of studies focused on the impacts that HPV may have on fertility and reproductive systems. This aspect of HPV infections deserves more attention because of the suggested association between HPV and reduced fertility or infertility [4]. The data supports the presence of HPV in semen and its proposed role in decreased fertility [2, 5]. It is worth mentioning that HPV DNA has been detected in endometrium and ovaries [6]. In a cohort study conducted in Denmark, no association has been found between high-risk HPV and the risk of female infertility [7], but in a review, it was concluded that HPV is associated with abnormalities in fertility and ART outcomes [8]. Another study revealed HPV-positive women were six times less likely to become pregnant after IUI [9]. Other studies found the detection of HPV at the time of fertility treatment has been adversely affected IVF outcomes (lower pregnancy rates and increased risk of early pregnancy loss) [10, 11]. Accordingly, HPV detection and genotyping in both men and women are suggested in infertility diagnosis, at least in idiopathic infertility cases, before IVF procedures [8]. Scientific literature indicates to high prevalence of genital HPV infection during pregnancy (around 40%) [12] and its association with adverse pregnancy outcomes such as spontaneous abortion, higher incidence of preterm pre-labor rupture of membranes (PPROM), and preterm birth [4, 7–9, 12]. Adequately, HPV positivity in pregnant women or their partners can be considered as a risk of miscarriages and premature rupture of membrane [4, 8]. Two studies indicated that the cervical conization and a LEEP do not necessarily increase the risk of preterm delivery in a subsequent pregnancy [13, 14]. Physiological changes during pregnancy and a decline in the functions of the immune system may increase the risk of oncogenic HPV persistence and progression to intraepithelial lesions in women older than 30 years of age [15]. Moreover, long-term use of birth control pills increases cervical cancer risk, importantly for women with persistent HPV infection. However, users of combined oral contraceptives (COCs) have a decrease in immune cells providing a favorable environment for the appearance of HPV lesions [16]. Women may worry about these issues but the literature is limited.

Tseng et al. found the overall frequency of HPV transmission from mothers to neonates was 39.7%, and a meaningfully higher rate of infection was observed when infants were delivered vaginally compared with cesarean [17]. There is still much controversy about the precise mode of HPV transmission to the fetus/ child. Detection of HPV DNA in semen, endometrium, and ovaries indicates the possibility of transmission even before conception [6, 18]. Another possible route of infection is intrauterine or prenatal transmission; because of the reported presence of HPV DNA in the amniotic fluid, placenta, and cord blood samples. Close contact of the fetus with the infected cervical and vaginal tracts of the mother during delivery can cause perinatal transmission. Maternal history of genital warts in pregnancy was associated with a higher risk of respiratory papillomatosis in the child [19]. Horizontal HPV transmission during breastfeeding or early nursing has also been considered as a significant contributor toward the infant's contagion. Inconsistent results have been found in terms of breast milk as a potential reservoir of viruses [6]. In light of lifelong HPV protection, considering the vaccination of infants is suggested [6].

To date, limited qualitative studies have explored adverse psychological responses to HPV diagnosis. Uncertainty about the psychological effects of a positive HPV test highlights the need for further research in this area [20]. Most of the reproductive concerns emerged from these studies. Few studies have addressed the fertility and pregnancy concerns of HPV-positive women. In a study conducted in the United States, it was reported that many women expressed fear associated with role of HPV in their future pregnancy [21]. In other studies, women expressed worries surrounding HPV and female subfertility [22–25]. One study addressed women's concern about the HPV-associated risks of preterm delivery and implications of natural delivery from an HPV-infected birth canal [22]. In another study women were worried about the HPV transmission to the fetus [26].

Given the effect HPV may have on women of reproductive age, the provision of support and interventions for infected women requires a deep understanding of their concerns. The current cervical cancer screening recommendation in Iran is co-testing (HPV testing and Pap smear) strategy for all women aged 30–59, with any marital status, every five years [27], which led to detecting many new cases of HPV in women of childbearing age [28]. Co-testing is available in all provinces of Iran although it is not covered by public insurance. Limited studies have mentioned reproductive concerns (often fears of infertility) in general. There have been no qualitative studies to investigate specifically reproductive concerns and informational needs of HPV-positive women particularly in an Islamic cultural background. Therefore, we conducted interviews with Iranian HPV-infected women to better understand their reproductive concerns.

## Methods

The design of the present study is qualitative. It was conducted based on the conventional content analysis approach to understand the reproductive concerns of HPV-positive women by exploring their feelings, experiences, and perceptions [29].

This study was carried out from September 2018 to December 2019 at the referral gynecology-oncology outpatient clinic of Valiasr (located in Imam Khomeini hospital complex, a large, busy, university-based, and geographically accessible complex in Tehran, founded in 1977) serving a large population of women from across the country (nearly 40 women daily). The clinic is equipped with colposcopy (two gynecology beds) and directed by oncologist-gynecologist SHSH (the last author) and her five colleagues.

A coordinator of Valiasr clinic sent all women who tested positive for HPV (either only high-risk HPV or both high-risk/low-risk strains) to the interviewer (KQ-female- no relationship with participants) in the calm, convenient room to provide them with information about the purpose and methods of the study. Women were eligible for interview if they were over 18 with a heterosexual partnership (with any marital status including: single, married, widow, divorced); had no severe disease (including cervical cancer) and were willing to share their experiences. A maximum variation purposive sampling was used to recruit information-rich candidates with diverse age, marital status, education, and socioeconomic status. In total, 20 Persian-speaking women with different ethnic, cultural, and religious backgrounds were included. Two invited women refused to participate because they prefer not to discuss HPV. Since the clinic is crowded, all participants interviewed during their waiting hours. Semi-structured one-to-one interviews were conducted using an interview guide (Appendix 1) started with the demographic background and reproductive and screening history. Three pilot-interviews were done (included in the study) to improve questions. Memos aided to design the next questions of the subsequent interviews. Besides, field notes were written during the interviews. Face-to-face in-depth interviews with participants' consent were recorded (lasted between 35 and 90 min), transcribed verbatim, and collected until data saturation was reached over fifteen months.

The data analysis was performed concurrently with data collection, using a qualitative content analysis approach described by Burnard et al. [30] using MAXQDA 10 software. Initially, interview transcripts, memos, and field notes were integrated, and two coders (KQ and STM) read the transcriptions multiple times to formulate a general understanding of the whole data. Open coding was based on this approach. Primary codes were then reduced by constant comparison and combination. The extracted codes were then brought together in terms of similarities and differences. The sub-categories with similar content were interpreted in a higher level of abstraction into the main categories.

The accuracy of this qualitative research was ensured according to the four criteria proposed by Guba and Lincoln, namely credibility, dependability, confirmability, and transferability [31, 32]. The credibility criterion was achieved through prolonged engagement and member checking, by which, the transcript and extracted codes from the interview were returned to each interviewee to approve their accuracy. Confirmability and dependability of the results were ensured by peer debriefing and external checking. Therefore, two observers reviewed and rechecked all transcripts, codes, and themes. Finally, this process completed with numerous discussions among the research team about areas of disagreement until reaching a final consensus. To enhance the transferability of the results, we tried to consider the maximum variation during sampling. We interviewed women with diversity in age, relationship status, education, socioeconomic status, and cultural background. In qualitative research, generalizability is labeled as a full description of the setting, the participants, and the themes in rich detail through the lens of the outside reader. To attain dependability, the process within the study was described in detail.

This study was undertaken as a part of a Ph.D. thesis in Reproductive Health, which was reviewed and approved by the Ethics Committee of Tehran University of Medical Sciences (IR.TUMS.FNM.REC.1397.139). Moreover, Valiasr hospital managers willingly facilitated the study. Written informed consent was obtained from all the participants. Direct quotes that are representative of the participants have been presented. Only the quotes used in the current manuscript have been translated into English.

## Results

The characteristics of 20 women interviewed demonstrate the heterogeneity of the sample (Table 1). Half of participants had children and 70 percent (14) were married. Participants averaged 33.9 years (aged 23–47). 70% of participating women had a university education. Details in parentheses following quotes represent the participant's identification number.

**Table 1:** Demographic characteristics, HPV genotypes and cytology results of participants

Participant ID number	Age (years)	Socio-economic level	HPV genotyping	Cervical cytology	Education	Occupation	Marriage duration (years)	Number of children	HPV duration (months)
1 Mina	32	High	High-risk	CIN-1	PhD	Lecturer	9	1	2
2 Eli	31	Middle	Mixed	CIN-1	Bachelor	Housewife	3	1	24
3 Razieh	30	Middle	High-risk	Normal	Master	Teacher	3	0	2
4 Inaz	28	High	Mixed	Normal	Bachelor	Employee	3	0	1
5 Goli	39	Middle	Mixed	CIN-2	Bachelor	Hair-dresser	10	1	24
6 Samira	32	Low	High-risk	ASC-US	Bachelor	Nurse	11	1	3
7 Nazanin	23	Low	Mixed	Normal	Bachelor	Student	2	0	12
8 Ida	30	Middle	High-risk	Normal	Diploma	Housewife	6	0	12
9 Bita	28	Low	High-risk	Normal	Bachelor	Student	Single	0	3
10 Mehri	37	Middle	Mixed	Normal	Diploma	Housewife	7	0	12
11 Hanieh	32	Low	High-risk	CIN-1	Bachelor	Employee	10	1	2
12 Shirin	33	Middle	High-risk	CIN-2	PhD	Employee	Single	0	6
13 Sara	44	Low	High-risk	ASC-US	Diploma	Housewife	23	3	13
14 Shadi	35	High	Mixed	ASC-US	Diploma	Housewife	Divorced	0	6
15 Kajal	47	Low	High-risk	CIN-2	High-School	Housewife	27	2	36
16 Donya	24	Low	Mixed	Normal	Bachelor	Shopkeeper	single	0	2
17 Flor	42	Middle	Mixed	ASC-US	Diploma	Housewife	20	2	12
18 Leila	41	High	High-risk	CIN-1	Master	Teacher	Widow	2	12
19 Neda	36	High	High-risk	CIN-2	Master	Employee	9	1	5
20 Bahar	35	High	High-risk	CIN-2	Bachelor	Musician	Divorced	1	1

*CIN* cervical intraepithelial neoplasia, *Mixed* both high-risk and low-risk HPV genotypes, *ASC-US* atypical squamous cells of undetermined significance

Many women reported seeking information about fertility and pregnancy from a range of sources, including the Internet (social media, blogs and websites run by private laboratories and specialists), their healthcare provider, and other women with HPV. They indicated that finding up-to-date trustworthy information was challenging. Most women preferred information provided by formal websites. Participants identified many fertility and childbearing concerns. The three main categories extracted from the interviews were "concerns about fertility potential," "pregnancy concerns," and "non-pregnancy reproductive concerns" (Table 2).

**Table 2:** Extracted categories and sub-categories from the interview data

Example of codes	Sub-categories	Categories
- Ambiguity about the effect of the virus on male fertility - Asking about the need for male GWs' treatment before conception	a. Adverse effects of HPV on male fertility	1. Concerns about fertility potential
- The effects of HPV on reproductive hormones and menstrual cycle - Concerns about infertility due to persistent high-risk HPV and subsequent cancer - Attributing the failure of assisted reproductive techniques (ART) to the HPV	b. Negative effect of HPV on female fertility	
- Fear of cervical infertility following invasive treatments (cryotherapy, LEEP, and conization) - The impact of the vaccine on hormones and the menstrual cycle - Fear of Infertility following HPV Vaccination - Concerns about the safety of HPV Vaccine in pregnancy	c. Threatened female fertility Association with treatments and vaccine	
- Concerns about increasing genital Warts during pregnancy - Worrying about weakening immune system during pregnancy - Limitation of performing diagnostic and therapeutic interventions (colposcopy-biopsy) in pregnancy - Postponing pregnancy for fear of progression of the disease	a. Threatened mother's health during pregnancy	2. Pregnancy concerns
- Fear of miscarriage due to HPV - Concerns about preterm birth due to HPV - Performing the cesarean section due to HPV	b. Adverse pregnancy outcomes	
- Fear of negative effects of GWs' medications on the fetus - Fear of transferring HPV to the fetus	c. Harm to the fetus	
- Worrying about infecting the infant via breast milk and nursing	a. Fear of infecting newborn	3. Non-pregnancy reproductive concerns
- Not using condom - Worries about the negative effect of Hormonal contraception on abnormalities - Need to a safe method of contraception	b. Concerns related to contraception method	
- Concerns about the effect of the virus on premature menopause	c. Fear of premature menopause	
- Attributing family history of gynecologic cancer to HPV	d. Fear of cervical cancer	
- Fear of cervical cancer following HPV infection	e. Fear of familial cancer due to HPV	

### Concerns about fertility potential

One of the most repeated concerns of HPV-positive women was fear of fertility impairment in both male and female patients mostly expressed by younger women who had pregnancy plans.

#### Adverse effects of HPV on male fertility

Some participants had questions about the presence of the virus in semen, sperm, and penis skin. They showed concerns about the effect of HPV on male fertility.

"We've been told to use a condom. Does this virus get into my husband's sperm? Isn't it weakening his fertility? I'm worried, it's because of this infection that I haven't got pregnant these years" (P.10).

Women with mixed HPV types whose husbands had genital warts (GWs) were mainly concerned about the importance of removing warts before conception.

#### Negative effect of HPV on female fertility

Participants were anxious and needed more information about the effect of papillomavirus on hormones and the reproductive system.

"You know why I'm worried? If I had a baby now, I wouldn't care about HPV. I'm 37. I have to get pregnant soon. I may lose my chance of getting pregnant. Does the virus affect my ovaries?" (P.10).

Some expressed fear and anxiety about losing their fertility due to getting cancer following persistent high-risk HPV.

I'm convinced that I am going to get cancer and I might never be able to have a baby in the future. I'm depressed because I'm not sure I can preserve my ability to get pregnant." (P.7).

Few women attributed the failure of assisted reproductive techniques (ART) to HPV.

"We've been trying to conceive for six months with no luck. We did IUI (Intra Uterine Injection) twice. I asked my doctor; she said the virus could be in the semen." (P.8).

#### Threatened female fertility association with treatments and vaccine

Women were concerned that therapeutic procedures (cryotherapy, loop electrosurgical excision procedure (LEEP), and conization) might impact their ability to get pregnant. A single woman who was about to have LEEP surgery for her cervical intraepithelial neoplasia (CIN)-2, expressed her concern as follows:

"I'm not sorry that I'm not getting married at all. I'm heartbroken that I don't know why, from the moment I find out [about HPV], I can feel … how much I wanted to have a baby. They (doctors) say there may not be a problem with this operation [LEEP], but there's. It can narrow the cervix and make it hard to get pregnant. All of those things run through your head." (P.12).

A wife planning to get pregnant after a LEEP asked:

"When can I start trying to conceive after a LEEP?" (P.19).

Few women were concerned about the potential effect of HPV vaccine on menstruation and fertility.

"I was spotting and lethargic every time I was vaccinated. Does HPV vaccine affect the period? I've read on Instagram that HPV vaccine can cause infertility" (P.14).

"Can Gardasil shots cause missed periods?" (P.16).

### Pregnancy concerns

Pregnancy for women infected with HPV had some challenges, mostly over personal health. Participants also expressed worries about fetal harm and adverse pregnancy outcomes including miscarriage, preterm delivery and cesarean section.

#### Threatened mother's health during pregnancy

Most HPV-positive women with abnormal cytology results were anxious that weakening the immune system during pregnancy could lead to the virus persistence in their body, and worsen cervical abnormalities. This fear was so great that some who had decided to become pregnant soon changed their pregnancy plans, postponing them until their cytology and HPV results return to normal.

"I wanted to get pregnant, so I went to a specialist for a checkup. Now that my test results [Pap, HPV, and colposcopy] came abnormal, I think it's not an appropriate time for me to get pregnant. I'm afraid pregnancy will make my results worse. I'm going to wait for the virus to go away, and then get pregnant." (P.1).

"I wanted to get pregnant. Then it happened [high-risk HPV and Atypical Squamous Cells of Undetermined Significance (ASC-US)], and I can't think of pregnancy anymore. My doctor said I could get pregnant, but I'm afraid I'll get pregnant, and my immune system will go down, and then my abnormal cells will grow." (P.6).

Some participants expressed concern about the safety of diagnostic and therapeutic procedures during pregnancy. They were worried that becoming pregnant would deprive them of timely treatment.

"Is colposcopy allowed in pregnancy? Could I be treated during pregnancy, if I get a serious precancerous condition?" (P.6).

Women with mixed HPV genotypes worried about increasing their warts during pregnancy. These thoughts discouraged and frustrated them.

"Pregnancy causes warts to multiply or get more noticeable. They're disgusting." (P.2).

They also had questions about preferred GWs treatments during pregnancy.

"If I get warts, what treatments are available for pregnant women? Can I freeze it?" (P.10).

#### Adverse pregnancy outcomes

Most women planning to have children in the future were concerned about the implications of infection on their (potential) child. women interviewed mentioned the association between genital HPV infection and various maternal and fetal variables and pregnancy complications such as miscarriage and premature delivery.

"I had a miscarriage last year. Was it from this virus? My Pap smear was always normal. What if I get pregnant and have a miscarriage again?" (P.4).

Women stated treatments like LEEP or conization might compromise their ability to carry a child to term by weakening the lining of the cervix.

"My sister's doctor removed abnormal cells from her cervix. After that, she became pregnant and her baby was born in the 35th week." (P.3).

Considering cesarean delivery for women with HPV was another matter posed by the participants. Some of them mistakenly believed that having genital warts was an indication of cesarean delivery to avoid perinatal development of laryngeal papillomatosis in the newborn.

"My doctor said if I had warts, I'd have a cesarean section. Thank god I did not have warts and I gave birth naturally." (P.2).

Misconceptions among healthcare professionals revealed in this regard which has caused concern among women.

#### Harm to the fetus

Fetal health was the main concern about pregnancy raised by most women interviewed. Participants also reported that they often found it challenging to find adequate information about this issue. They needed to know what to do to protect their child from the infection. In this regard, one of the participants planning to get pregnant soon stated:

"I worry about passing HPV to the baby during pregnancy and childbirth. What would I do to stop that?" (P.4).

Since anogenital warts can proliferate during pregnancy, removal of warts during pregnancy was another issue that was raised. Women with GWs revealed information needs surrounding the teratogenicity of some wart-removing treatments. They felt anxious about any threats that might pose to the fetus.

"Doctor gave me Podophyllin, saying I shouldn't get pregnant while using it. I'm afraid I'm pregnant. I want to know which wart-removing medicine is safe in pregnancy." (P.4).

They also wanted to know if intercourse during pregnancy would increase the chance of HPV transmission to the fetus.

Almost all women interviewed reported being advised to take the HPV vaccine. Participants knew little about the safety of the HPV vaccine (mostly Gardasil) in pregnancy. Women who wanted to get pregnant preferred not to become pregnant until they completed the vaccine series.

"I asked about vaccination and pregnancy. Doctor recommended postponing vaccination until after pregnancy. But I'd rather to get vaccinated first." (P.2).

## Non-pregnancy reproductive concerns

Some reproductive concerns unrelated to pregnancy have reported in the third category. Five challenges discussed in this category were breastfeeding, contraception method, premature menopause, cervical cancer, and familial cancer.

### Fear of infecting newborn

Two women mentioned the likelihood of passing HPV to a child through breast milk or early nursing.

"Does HPV affect breast milk, I mean, just like a diet? Does breastfeeding cause mouth warts? Should we avoid breastfeeding?" (P.3).

### Concerns related to contraception method

To avoid unplanned pregnancies, women with HPV needed more information to choose preferable contraception. As long-term use of birth control pills increases the cervical cancer risk for women with persistent HPV, users recommended changing their contraception method. Women were worried about the negative impacts of combined oral contraceptives (COCs) and levonorgestrel (LNG) pills on their cellular changes.

"We use the pull-out (withdrawal) to prevent pregnancy, but sometimes I use emergency pills. I don't know if they can weaken my immune system. I read online that birth pills may induce cervical cancer. Are the emergency pills as harmful as the COCs?" (P.17).

"I've taken LD (low dose COC) pills after my daughter was born, which is about nine years. They said I have to stop taking them. I don't know what to do. My husband does not use a condom." (P.11).

Some women in a monogamous relationship reported they (or their husbands) are reluctant to use a condom. They wanted to know why using a condom is essential for HPV-positive patients when they already had HPV.

### Fear of premature menopause

A few women over 40 who had no pregnancy intention mentioned concerns about early menopause. Testing positive for high-risk HPV genotypes and abnormal cytology have made these participants fearful of premature ovarian insufficiency.

"I have noticed my periods becoming infrequent. I've tested positive for HPV 16. I feel that my periods get missed and irregular due to this disease. Could this virus cause me to menopause? I'm worried I'm in menopause. I don't like to get there." (P.18).

Another stated: "I had a hysterectomy last year (because of fibroids and heavy periods). They hadn't removed my ovaries. I also had a high-risk HPV 53, but I got vaccinated, I'm worried about the effect of HPV on my ovaries." (P.15).

### Fear of cervical cancer

One of the most common concerns of women tested positive for HPV was cervical cancer that has described in a different manuscript as the psychological response to HPV diagnosis. A context-specific finding that seems specific to societies that adhere to cultural principles was fear of cervical cancer in virgin single women. Since vaginal virginity is a matter of prestige in most parts of Iran, they reported engaging in sexual intercourse without vaginal penetration. Two virgin women have expressed concern about cervical cancer following an ascending HPV infection from the perineum to the cervix. After getting genital warts and learning about HPV-related cancers, they were worried about cervical cancer. One revealed that:

"You don't know what is going on because you can't take Pap smear or colposcopy. I was scared. My gynecologist performed so-called Girly Pap smear. She took a cotton swab sample from the end of the vagina for HPV typing and cytology." (P.16).

### Fear of familial cancer due to HPV

Few women were anxious about the possible association between HPV and history of cancer in their female family members.

"My sister had a mastectomy last year. I wonder her cancer was associated with HPV. We both have HPV. I'm scared what if it's a familial thing, and then I might get cancer too." (P.9).

Another woman with CIN-2 and high-risk types mentioned:

"My mother died of cancer. Did it have anything to do with HPV?" (P.20).

## Discussion

The present study aimed to shed some light on the reproductive concerns of women infected with HPV and found they have reproductive concerns and informational needs over the effects of HPV infections on male and female fertility potential, the success of ART techniques (including IVF and IUI), fetal and newborn health, mother health and pregnancy outcomes. Our participants had questions and worries about the presence of HPV in the semen and the impact of HPV infection upon male fertility. Although concerns over male subfertility in HPV-positive men has received far less attention in previous qualitative studies, concerns about female fertility were generally mentioned in previous studies [23–25, 27, 33, ﻿34﻿]. Our findings highlighted reproductive concerns in more details as mentioned above.

One study, conducted in Lithuania, has identified HPV-52 as the most common form of HPV in couples undergoing IVF1. Interestingly, the two participants who expressed the most concern over infertility were diagnosed with HPV-52. We believe more research should be conducted on the relationship between HPV-52 and infertility. Worries about the failure of ART procedures, including IVF and IUI, due to HPV infections do not seem to be unfounded, according to literature [9–11]. Health care providers need to take these concerns seriously and consider referring women to specialized level.

Fertility fears about the HPV-vaccination among women diagnosed with HPV have been reported in a qualitative study [17]. Our participants expressed their fear of losing fertility after getting HPV vaccine. It can be due to considerable media attention to the safety of the HPV vaccine. World Health Organization (WHO) has cited to a systematic review that concluded no causal relations between HPV vaccination and infertility [35]. In the present study, the participants feared that the accidental injection of the HPV vaccine during pregnancy would cause adverse pregnancy outcomes or harm to the fetus. Women should be informed by healthcare providers that although pregnancy testing is not necessary before the vaccination, the vaccine manufacturers and WHO recommend avoiding HPV vaccination during pregnancy. In cases of unintentional immunization of pregnant women, no intervention is needed [36, 37].

In agreement with other studies, our participants mentioned fertility concerns about conservative treatment for CIN like LEEP and conization [22, 24, 34]. Research has yielded mixed results, but one study indicates that fertility is not affected by a LEEP [38].

Pregnancy concerns were the second category extracted from the interview data. Women mentioned that worrying about the adverse effects of pregnancy on their health was the main reason they decided not to get pregnant. In a mixed-method study conducted in the United States, 30 of 94 women who planned on getting pregnant indicated that their HPV test results would change their future pregnancy plans [39]. Women infected with HPV needed to know what they need to do to have a healthy pregnancy. They may also face barriers seeking and obtaining information to address their reproductive concerns [36].

In line with our findings, two studies also indicated concerns about the association between HPV infections and adverse pregnancy outcomes such as spontaneous abortion and preterm delivery [22, 34]. In conjunction with Pourmohsen's study, maternal-to-fetal HPV transmission was a common concern, especially among married who had the pregnancy plans. Women interviewed thought natural childbirth may pose a transmission risk to a newborn. Another qualitative study reported this concern [22]. Although there is still controversy about adverse effect of HPV on pregnancy outcomes, women may worry about these issues and physicians need to address these concerns. Most participants reported being highly concerned about the safety of wart-removing medications and diagnostic and therapeutic interventions for cervical cell changes during pregnancy. They asked about the safest way to remove GWs. In a study, CO2 laser vaporization has been suggested as a safe, simple treatment for warts during pregnancy [40].

Almost all interviewed women had been recommended to use a condom. Most married reported that they (or their husbands) are reluctant to use a condom. They wanted to know why constantly using condoms is essential while they already are HPV-infected. Caregivers should also explain why they recommend using a condom.

Some women with a history of taking hormonal contraceptives pointed out they have been recommended to stop taking combined hormonal pills. HPV-positive women raise some concerns about choosing their contraceptive method. Although, to conclude causality between COCs and HPV lesions, more studies are needed [16], particular attention should be given to discuss contraception methods with HPV-positive women.

Such richness from the interviews revealed unexpected concerns such as fear of having an ovarian tumor and getting cancer because of a family history of cancer. They considered HPV to be a familial carcinogen factor. Similar concerns indicated by another study [41].

The only context-specific finding was the fear of cervical cancer in single virgin women. Communities define 'virginity' in the different ways. In our society intact hymen determines someone's virginity. In this Islamic-Iranian context HPV-positive virgin women avoid vaginal examination. To reassure these women, doctors take a so-called "Girly Pap smear" from the upper part of the vagina with a cotton swab. Our findings revealed misconceptions regarding the relationship between non-penetrative sex and STIs among single women. These women still followed up with the gynecologist but did not have a proper pap-smear and hence may miss out on the opportunity of being diagnosed properly.

In previous studies, the reproductive concerns of HPV-positive people had been discussed as a part of emotional and psychological responses to HPV diagnosis. Our findings are noteworthy because as far as we are aware, this is the only qualitative study in which reproductive concerns of HPV-positive women have received particular attention. Furthermore, women with diversity in HPV genotypes (both high-risk and low-risk) were interviewed. As queries about sexual health were part of the interview content, they will be discussed in another manuscript entitled "Sexual life of HPV-positive women." One of the limitations is that the study was conducted among women attending one colposcopy clinic. However, this referral clinic is likely to reflect other clinics in Iran since it covers a varied population. Possibly women who were interested in the topic decided to participate, and it applies to all qualitative research. We should comment on the possible impact of having a highly educated sample (~ 70% with university education) as another limitation. Unlike previous studies that women with genital warts are often excluded, a subset of our participants was HPV-positive women who had GWs. It is worth noting that these women may have additional concerns that must not be overlooked. Moreover, the relative weight or importance of themes and categories is not always apparent. The credibility of the process is demonstrated in the sentiments stated in the data set, suggesting that the extracted concerns may be transferrable to other settings. An additional strength is that this paper complies with the COREQ checklist designed for the reporting of qualitative studies [42].

## Conclusions

The findings of this qualitative study suggest HPV-positive women's concerns about the possible effect of HPV on male and female fertility, pregnancy outcomes, mother and child health, breastfeeding and contraceptive methods. Most of these concerns were based on actual proven correlations and should be addressed. These concerns need to be taken into account when Health care providers are counseling HPV-positive women. Medical professionals need to take women's informational needs more seriously to become one step closer to helping these women improve their reproductive health. Extracted concerns which are based on misinformation, such as recommending C-section for women with GWs or fear of HPV vaccine, require increasing education /awareness for patients and/or healthcare providers. Moreover, health care providers could benefit from additional training to be prepared to mitigate HPV-positive women's reproductive concerns. Women need to have better informational resources about these sensitive topics so that they can make informed decisions about having children. Some women may also benefit from referral to a specialist in the context of assisted reproduction.

## Data Availability

The datasets used and/or analysed during the current study are available from the corresponding author on reasonable request.

## Appendix 1: Interview guide

"What do you know about HPV?", "What have you been told or what do you know about the effects of HPV on the reproductive system of both men and women?", "Please tell me about your experiences, thoughts, and feelings regarding pregnancy and childbearing?", "Are you experiencing any concerns?", "How do you describe pregnancy and motherhood while infecting with HPV?", "Is your personal pregnancy plan affected by the diagnosis? And how?", and "What you need to know as a woman infected with HPV about your reproductive health (menstrual cycle, marriage, contraception, pregnancy, breastfeeding, menopause, and gynecological cancers)?", "How you seek for answer to your questions?" and "Which source of information do you prefer? And why?" Open questions such as "What do you mean?" and "Please expand" were used to extract more clear and detailed responses.

## Abbreviations

HPV: Human Papilloma Virus 1
UNFPA: United Nations Fund for Population Activities
WHO: World Health Organization
GWs: Genital warts
ART: Assisted reproductive techniques
IUI: Intra uterine injection
LEEP: Loop electrosurgical excision procedure
CIN: Cervical intraepithelial neoplasia
ASC-US: Atypical squamous cells of undetermined significance
PPROM: Preterm pre-labor rupture of membranes
COCs: Combined oral contraceptives
LD: (Low dose) combined oral contraceptives pills
LNG: Levonorgestrel
IVF: In vitro fertilization
STIs: Sexually transmitted infections

## References

[1] Centre IIHI. Iran: human papillomavirus and related cancers, fact sheet. 2018. ICO/IARC HPV Information Centre, 2018.

[2] Jersoviene V, Gudleviciene Z, Rimiene J, Butkauskas D (2019). Human papillomavirus and infertility. Medicina.

[3] Souho T, Benlemlih M, Bennani B (2015). Human papillomavirus infection and fertility alteration: a systematic review. PLoS ONE.

[4] Xiong YQ, Chen YX, Cheng MJ, He WQ, Chen Q (2018). The risk of human papillomavirus infection for male fertility abnormality: a meta-analysis. Asian J Androl..

[5] Capra G, Schillaci R, Bosco L, Roccheri MC, Perino A, Ragusa MA (2019). HPV infection in semen: results from a new molecular approach. Epidemiol Infect..

[6] Merckx M, Liesbeth W-VW, Arbyn M, Meys J, Weyers S, Temmerman M (2013). Transmission of carcinogenic human papillomavirus types from mother to child: a meta-analysis of published studies. Eur J Cancer Prev..

[7] Nohr B, Kjaer SK, Soylu L, Jensen A (2019). High-risk human papillomavirus infection in female and subsequent risk of infertility: a population-based cohort study. Fertil Steril.

[8] Zacharis K, Messini CI (2018). Human papilloma virus (HPV) and fertilization: a mini review. Medicina..

[9] Ambühl LM, Baandrup U, Dybkær K, Blaakær J, Uldbjerg N, Sørensen S (2016). Human papillomavirus infection as a possible cause of spontaneous abortion and spontaneous preterm delivery. Infect Dis Obstet Gynecol..

[10] Perino A, Giovannelli L, Schillaci R, Ruvolo G, Fiorentino FP, Alimondi P (2011). Human papillomavirus infection in couples undergoing in vitro fertilization procedures: impact on reproductive outcomes. Fertil Steril..

[11] Spandorfer SD, Bongiovanni AM, Fasioulotis S, Rosenwaks Z, Ledger WJ, Witkin SS (2006). Prevalence of cervical human papillomavirus in women undergoing in vitro fertilization and association with outcome. Fertil Steril.

[12] Pandey D, Solleti V, Jain G (2019). Human papillomavirus (HPV) infection in early pregnancy: prevalence and implications. Infect Dis Obstetr Gynecol.

[13] Andía D, Mozode Rosales F, Villasante A, Rivero B, Díez J, Pérez C (2011). Pregnancy outcome in patients treated with cervical conization for cervical intraepithelial neoplasia. Int J Gynecol Obstetr..

[14] Ehsanipoor RM, Jolley JA, Goldshore MA, Szymanski LM, Haydon ML, Gaffaney CL (2014). The relationship between previous treatment for cervical dysplasia and preterm delivery in twin gestations. J Maternal-Fetal Neonatal Med.

[15] Hernández-Girón C, Smith JS, Lorincz A, Lazcano E, Hernández-Avila M, Salmerón J (2005). High-risk human papillomavirus detection and related risk factors among pregnant and nonpregnant women in Mexico. Sex Transm Dis..

[16] Volpato LK, Siqueira IR, Nunes RD, Piovezan AP (2018). Association between hormonal contraception and injuries induced by human papillomavirus in the uterine cervix. Revista Brasileira Ginecologia Obstetricia.

[17] Tseng CJ, Liang CC, Soong YK, Pao CC (1998). Perinatal transmission of human papillomavirus in infants: relationship between infection rate and mode of delivery. Obstetr Gynecol..

[18] Skoczyński M, Goździcka-Józefiak A, Kwaśniewska A (2019). Co-occurrence of human papillomavirus (HPV) in newborns and their parents. BMC Infect Dis..

[19] Pradhan SR, Mahata S, Ghosh D, Sahoo PK, Sarkar S, Pal R (2020). Human papilloma virus infections in pregnant women and its impact on pregnancy outcomes: possible mechanism of self-clearance.

[20] Bennett KF (2019). The psychosexual impact of testing positive for high-risk cervical human papillomavirus (HPV): a systematic review. Turk J Obstet Gynecol..

[21] Perrin KK, Daley EM, Naoom SF, Packing-Ebuen JL, Rayko HL, McFarlane M (2006). Women’s reactions to HPV diagnosis: insights from in-depth interviews. Women Health..

[22] Kosenko KA, Hurley RJ, Harvey JA (2012). Sources of the uncertainty experienced by women with HPV. Qual Health Res..

[23] Waller J, McCaffery K, Kitchener H, Nazroo J, Wardle J (2007). Women’s experiences of repeated HPV testing in the context of cervical cancer screening: a qualitative study. Psychooncology..

[24] McCaffery K, Waller J, Nazroo J, Wardle J (2006). Social and psychological impact of HPV testing in cervical screening: a qualitative study. Sex Transmit Infect..

[25] Daley EM, Perrin KM, McDermott RJ, Vamos CA, Rayko HL, Packing-Ebuen JL (2010). The psychosocial burden of HPV: a mixed-method study of knowledge, attitudes and behaviors among HPV+ women. J Health Psychol.

[26] Pourmohsen M, Simbar M, Nahidi F, Fakor F, Majd HA (2020). Women’s experiences of infection with human papillomavirus in the face of disease symptoms: a qualitative study. Int. J. Womens Health Reprod. Sci..

[27] Kousha AMA, Maleki A, Najmi M, Dini M, Arjmandpour M (2017). Package of essential non-communicable (PEN) disease interventions for primary health care in lran “IRAPEN”.

[28] Bruni LB-RL, Albero G, Serrano B, Mena M, Gómez D, Muñoz J, Bosch FX, de Sanjosé S. ICO information centre on HPV and cancer (HPV information centre). Hum Papillomavirus Relat Dis Iran. 2017.

[29] Hsieh H-F, Shannon SE (2005). Three approaches to qualitative content analysis. Qual Health Res.

[30] Burnard P, Gill P, Stewart K, Treasure E, Chadwick B (2008). Analysing and presenting qualitative data. Br Dent J.

[31] Polit DF, Beck CT (2008). Nursing research: generating and assessing evidence for nursing practice.

[32] Lincoln YS, Guba EG (1986). But is it rigorous? Trustworthiness and authenticity in naturalistic evaluation. New Direct Program Eval.

[33] Graziottin A, Serafini A (2009). HPV infection in women: psychosexual impact of genital warts and intraepithelial lesions. J Sex Med.

[34] McRae J, Martin C, O'Leary J, Sharp L (2014). “If you can't treat HPV, why test for it?” Women’s attitudes to the changing face of cervical cancer prevention: a focus group study. BMC Women’s Health..

[35] who.int. Human papilloma virus vaccines and infertility WHO Weekly Epidemiological Record of 24 January 2020; 2019. Extract from GACVS meeting of 4–5 December 2019]. https://www.who.int/vaccine_safety/committee/topics/hpv/Dec_2019/en./. Accessed Jul 2020.

[36] Bonde U, Joergensen JS, Lamont RF, Mogensen O (2016). Is HPV vaccination in pregnancy safe?. Hum Vaccin Immunother..

[37] Tan J, Xiong YQ, He Q, Liu YM, Wang W, Chen M (2019). Peri-conceptional or pregnancy exposure of HPV vaccination and the risk of spontaneous abortion: a systematic review and meta-analysis. BMC Pregnancy Childbirth..

[38] Kyrgiou M, Mitra A, Arbyn M, Paraskevaidi M, Athanasiou A, Martin-Hirsch PP (2015). Fertility and early pregnancy outcomes after conservative treatment for cervical intraepithelial neoplasia. Cochrane Database Syst Rev..

[39] Daley EM, Marhefka SL, Buhi ER, Vamos CA, Hernandez ND, Giuliano AR (2010). Human papillomavirus vaccine intentions among men participating in a human papillomavirus natural history study versus a comparison sample. Sex Transm Dis..

[40] Savoca S, Nardo LG, Rosano TF, D’Agosta S, Nardo F (2001). CO(2) laser vaporization as primary therapy for human papillomavirus lesions. A prospective observational study. Acta Obstetr Gynecol Scand..

[41] Lin H, Jeng C-J, Wang L-R (2011). Psychological responses of women infected with cervical human papillomavirus: a qualitative study in Taiwan. Taiwan J Obstet Gynecol.

[42] Tong A, Sainsbury P, Craig J (2007). Consolidated criteria for reporting qualitative research (COREQ): a 32-item checklist for interviews and focus groups. Int J Qual Health Care..

